# mHealth for Tuberculosis Treatment Adherence: A Framework to Guide Ethical Planning, Implementation, and Evaluation

**DOI:** 10.9745/GHSP-D-16-00018

**Published:** 2016-06-20

**Authors:** Michael J DiStefano, Harald Schmidt

**Affiliations:** a University of Pennsylvania, Department of Medical Ethics and Health Policy, Philadelphia, PA, USA; b University of Pennsylvania, Department of Medical Ethics and Health Policy, Center for Health Incentives and Behavioral Economics, Philadelphia, PA, USA

## Abstract

Promising mHealth approaches for TB treatment adherence include:

- Video observation
- Patient- or device-facilitated indirect monitoring
- Direct monitoring through embedded sensors or metabolite testing

To mitigate ethical concerns, our framework considers accuracy of monitoring technologies, stigmatization and intrusiveness of the technologies, use of incentives, and the balance of individual and public good.

## INTRODUCTION

Adherence to tuberculosis (TB) treatment is important for promoting individual and public health. Poor adherence results in more individual suffering and death as well as more costly treatment as treatment regimens lengthen and drug resistance develops. As resource allocation pressures intensify under the Sustainable Development Goals and the global push for universal health coverage,^1^ exploring novel ways of improving adherence is timely and important.

Mobile health (mHealth) holds considerable promise to improve quality and efficiency in health care.^2^ Yet its potential for TB adherence remains largely unexplored,^3^^,^^4^ and there is a lack of people-centered mHealth approaches responsive to the complexity of real life.^5^ Also lacking are ethical evaluations of mHealth interventions,^6^^,^^7^ particularly those considering the nuances of disease- and goal-specific interventions. Perceived and real acceptability cannot be taken for granted. The planning, implementation, and evaluation of mHealth interventions for TB treatment adherence must be guided by values as much as by technical innovation.

mHealth interventions for TB treatment adherence must be guided by ethical values as much as by technical innovation.

In this article, we describe salient mHealth approaches to monitor and enhance TB treatment adherence, establish a framework for consideration of the central ethical issues, particularly when mHealth is paired with incentives, and outline a model to help guide the ethical planning, implementation, and evaluation of future mHealth interventions for adherence. In doing so, we highlight areas of ethical concern as well as opportunities for ethical improvement over direct observation of therapy (DOT), the global standard for monitoring TB treatment adherence.

## TUBERCULOSIS AND THE IMPORTANCE OF ADHERENCE

In 2014, 9.6 million people became ill with TB and 1.5 million died, ranking TB alongside HIV as a leading cause of death worldwide.^8^ More than 95% of TB deaths occur in low- and middle-income countries (LMICs).^9^

According to the World Health Organization (WHO), the optimal TB treatment plan consists of an initial treatment phase requiring daily ingestion of 4 first-line anti-TB drugs for 2 months, followed by a 4-month continuation phase during which 2 daily drugs are necessary. Regimens of ingesting drugs thrice weekly in both the initial and continuation phase are also possible.^10^ TB susceptible to first-line drugs is called drug-susceptible TB (DS-TB). Two forms of drug-resistant TB (DR-TB) are widely recognized: multidrug-resistant TB and extensively drug-resistant TB. Treating even the less resistant forms of DR-TB can take up to 2 years and may also require daily medication.

Proper treatment of all forms of TB is critical to reducing individual morbidity and mortality and to interrupting transmission among family and community members. Proper treatment also limits the development and spread of DR-TB. Treatment for DR-TB is more expensive, less effective, and has more serious side effects.^11^ The impact of poor treatment on morbidity, mortality, and disease transmission is exacerbated by poverty, weak health systems, and low levels of health literacy found in many LMICs. Because proper treatment rests in part on proper adherence, monitoring and enhancing adherence is important to safeguarding both individual and public health.

Proper TB treatment rests in part on proper patient adherence, so monitoring and enhancing adherence is critical.

Ensuring adherence is therefore a key component of WHO’s post-2015 global TB strategy—the “End TB Strategy.” Pillar 1 of this strategy calls for “supportive treatment supervision … [to help] patients to take their medication regularly and to complete treatment, thus facilitating their cure and preventing the development of drug resistance.”^12^ The strategy indicates supervision should be “carried out in a context-specific and patient-sensitive manner” and acknowledges the many barriers to adherence, including “educational, emotional, and material needs,” “stigmatization and discrimination,” and health-system factors^12^ Given these diverse barriers, supportive treatment supervision will be necessarily complex, spanning interventions to train treatment partners, provide greater social protection, disseminate and exchange necessary information and experiences across potentially long distances, and using incentives.

Where supportive treatment supervision includes monitoring of adherence, DOT (i.e., when a health worker directly witnesses the swallowing of anti-TB drugs in a clinical, community, work, or personal setting) has long been recommended by WHO and is the global standard of care. New adherence monitoring techniques should therefore be compared with DOT in terms of ethical acceptability.

New TB treatment adherence monitoring techniques should be compared with DOT in terms of ethical acceptability.

### Barriers to Adherence

While DOT has seen great success in specific contexts,^13^ its limitations can be understood against the barriers of non-adherence recognized in WHO’s End TB Strategy. Munro et al. elaborated on these barriers by identifying and describing 4 categories of adherence barriers related to structural, patient, social, and health care service factors.^14^ The influence and interplay of these 4 factors vary. Addressing non-adherence to TB treatment therefore requires awareness of context and targeting all relevant factors.

Barriers to TB treatment adherence can be many, relating to structural, patient, social, and health care service factors.

#### Structural Factors

Structural factors are obstacles, such as poverty and gender, over which patients have very little control and which can complicate adherence even when patients are strongly motivated. Poverty, especially when linked to the factors discussed below, can impact adherence. For example, where treatment costs are not covered, poor patients or those supporting others, may feel they must choose between work and health. In LMICs, the mean total cost of TB (i.e., direct medical and non-medical costs and lost income due to sickness) is 39% of annual reported household income, and 148% at its highest.^15^ Gender-related factors may also impact adherence. TB-related stigma can lead to greater concealment and denial of disease among women than men, and gender roles within traditional or poor families that translate to women having less leisure time and status may result in greater difficulties for women to pursue treatment.^16^

#### Patient Factors

Variations in patient motivation and willingness can also impact adherence and may be affected by forgetfulness, a lack of understanding regarding the importance of TB treatment, general interpretations of illness such as the belief that one is sick only if symptomatic, alcohol or drug use, or a perceived loss of agency and aversion to elements of control and surveillance associated with DOT. Furthermore, side effects of TB drugs, which include fever, fatigue, weakness, nausea, vomiting, hepatitis, or death,^17^ can affect patient motivation due to unpleasantness or substantial interference with a patient’s ability to work. This is especially true as the side effects grow more diverse and severe during DR-TB treatment to include psychiatric disorders, hearing loss, and epileptic seizures.^18^

#### Social Context

Strong social support within a patient’s family, community, or health care context can help counteract structural and personal barriers to adherence by influencing motivation or knowledge and beliefs about TB. However, lack of support or knowledge about TB and its treatment in a patient’s family, community, or health care context, as well as real or perceived stigmatization of the sick, can hinder adherence.

#### Health Care Service Factors

Inadequate drug stocks, long waiting times, inconvenient service hours, and difficulties accessing health facilities reveal the opportunity costs associated with attending health facilities, such as neglecting household responsibilities (e.g., caring for one’s children) and losing work and income. All these factors can therefore thwart adherence to TB treatment. For example, a study in Ethiopia found that the average patient traveled approximately 70 hours to a DOT health facility. Following the initial treatment phase, patients traveled only once per month to a DOT health facility to collect drugs. Distance rendered regular observation of drug ingestion impractical (either on a daily or alternating daily basis) and may have contributed to treatment success rates that fell below WHO targets.^19^

## THE PROMISE OF MHEALTH FOR TB TREATMENT ADHERENCE

While a multitude of barriers to TB treatment adherence needs to be considered, mHealth interventions can potentially address several central adherence challenges. First, the number of mobile cellular subscriptions per 100 people in LMICs is 87 and growing^20^: mHealth can potentially obviate DOT-related travel and improve adherence in remote areas. Second, mHealth may improve health system efficiency in regions where resources and trained medical professionals are very scarce. For instance, in South Africa, an intervention that relies on mobile phones and SMS (short message service) has enabled nurses to track 50 to 60 TB patients instead of only 10, thus making time to focus on otherwise neglected aspects of their work.^21^

Additionally, mHealth can address important patient factors in non-adherence by facilitating novel and sophisticated ways of providing financial and non-financial incentives. For example, a study of warfarin adherence used pill compartments that wirelessly entered patients into a daily lottery when opened according to the prescribed treatment plan,^22^ therefore eliminating the need for human observation and recordkeeping. Mobile platforms can also help overcome some logistical challenges of incentive delivery. Examples from Kenya,^23^ Malawi,^24^ and Zambia^24^ demonstrate the feasibility of efficient electronic transfer schemes that eliminate the need for travel to a bank or remote patient areas, potentially expanding the reach of incentive programs, lowering the costs of incentive delivery, and rendering the process more sustainable.

mHealth can facilitate novel ways of providing financial and non-financial incentives to encourage TB treatment adherence.

Finally, empirical research supports mHealth’s promise for improving adherence. A recent study, the first to conduct a large and rigorous trial in this area, found that electronic reminders from medication monitors improved TB treatment adherence.^25^ Various smaller, proof-of-concept studies have established the potential benefit of using other forms of mHealth for TB adherence.^4^

## AN ETHICAL FRAMEWORK

We conducted a comprehensive search of PubMed and Google Scholar on March 15, 2016, for mHealth interventions focused on TB adherence using the following 2 search strings:

- “adherence AND (eHealth OR mHealth OR ‘electronic health’ OR ‘mobile health’ OR e-health OR m-health)”
- “adherence[Title/Abstract]) AND "systematic review"[Title/Abstract]) AND (eHealth OR mHealth OR ‘electronic health’ OR ‘mobile health’ OR e-health OR m-health)”

Adapting and adding to previously identified types of mHealth for TB adherence,^26^ we constructed the following 5 intervention categories (Table 1):

1. **Video observation of therapy (VOT):** patients use smartphones to record videos of themselves taking each medication dose, allowing health care workers to view the videos either synchronously or asynchronously; facial recognition and motion-detecting software can even replace the need for human observation
2. **Indirect monitoring technology, patient-facilitated (IP):** after ingesting their medication, patients place a free call or send an SMS to a central server
3. **Indirect monitoring technology, device-facilitated (ID):** after the patient removes the cap of the medication bottle, a message is wirelessly transmitted to a central server
4. **Direct monitoring technology, embedded sensors (DE):** when the patient ingests the medication, which is equipped with an ingestible sensor, a wearable hub attached to the patient’s body wirelessly transmits the data to a central server
5. **Direct monitoring technology, metabolite testing (DM):** patients use low-cost, encrypted chromatography urine test strips, which detect drug metabolites in the patients’ urine revealing a code; the patients then send an SMS with the code to a central sever

**TABLE 1:** Types of mHealth Interventions for TB Treatment Adherence Compared With the Global Standard of Direct Observation of Therapy

Intervention Category	Examples	Description
Direct observation of therapy (DOT) – Global standard	WHO DOTS^27^	A DOT worker directly observes and records patient swallowing medication.
Video observation of therapy (VOT)	VDOT System^28^	Patients use smartphones to record videos of themselves taking each dose of medication. Observation by a health official can be either synchronous (“video conferencing”) or asynchronous (“store-and-forward”).^28^ Other systems, such as the Automated DOT software, replace human observation with facial recognition and motion-detecting software.
	Automated DOT^29^	
Indirect monitoring technology		
Patient- facilitated (IP)	SIMmed^21^	Patients place a free call or send an SMS to a central server after ingesting medication.
	99dots^30^	
Device- facilitated (ID)	SIMpill^21^	Medication bottles containing a SIM card or other sensor wirelessly transmit message to a central server following medication cap removal by a patient.
	MEMSCap^31^	
	GlowCap^32^	
	Wisepill^33^	
Direct monitoring technology		
Embedded sensors (DE)	ID-cap^34^	A wearable hub that attaches to the patient’s wrist, arm, hip, or abdomen detects when the patient ingests medication or a pill capsule, equipped with an ingestible sensor, and wirelessly transmits the data to a central server.
	Proteus^35^	
Metabolite testing (DM)	Adhere.IO^36^	Patients receive low-cost, encrypted chromatography test strips for home use, which reveal codes when correct drug metabolites are detected in the patient’s urine. The patient sends an SMS with the code to a central server.

Abbreviations: DOTS, directly observed treatment, short-course; SIM, subscriber identification module; SMS, short message service; TB, tuberculosis; WHO, World Health Organization.

In our analysis, we considered the possibility that any mHealth intervention for adherence can comprise, first, a monitoring technology and, second, features that use monitoring data to enhance adherence, such as personal reminders or incentives. We discuss the potential of the 5 categories of mHealth interventions to ethically monitor and enhance TB treatment adherence in line with WHO’s “supportive treatment supervision” guidelines, and in comparison with DOT—the global standard of care for adherence monitoring. Building on prior work that analyzed the ethics of policies to promote individual responsibility for health,^37^ we developed an analytical framework for this comparison that comprises 4 ethically relevant elements:

1. Accuracy of monitoring technologies
2. Stigmatization and intrusiveness of monitoring technologies
3. The use of incentives
4. The balance of individual and public good

Any mHealth adherence intervention can comprise (1) a monitoring technology and (2) features that use monitoring data to enhance adherence.

In discussing strengths and weaknesses of each mHealth intervention category, we identify areas of ethical concern as well as opportunities for ethical improvement over DOT. Finally, we outline a model using the 4 ethical elements to help guide the ethical planning, implementation, and evaluation of future mHealth interventions for TB adherence (Table 2). This recommended model will always require adaptation to suit specific contexts—for example, the relative prevalence of DS-TB versus DR-TB in resource-limited settings and the different challenges presented by varying levels of government oversight—but hopefully provides useful baseline orientation.

**TABLE 2:** Framework for the Ethical Evaluation of DOT and mHealth Interventions for TB Treatment Adherence, by Deceasing Accuracy of Adherence Detection

Intervention		Criteria for Ethical Evaluation			
(1) Monitoring Technology	(2) Feature(s) to Enhance Patient Adherence	(1) Accuracy	(2) Stigmatization and Intrusiveness	(3) The Use of Incentives	(4) Balance of Individual and Public Good
**Direct monitoring technology (metabolite testing) (DM)**	Follow-up by health care workers when non-adherent SMS reminders to take medication throughout treatment Reward incentives (e.g., in-kind goods, or reductions in insurance contributions)^a^ conditional on good adherence Penalty incentives (e.g., insurance surcharges) when non-adherent	**Most accurate:** medication ingestion and metabolization is necessary for adherence detection	**Least risk:** urinalysis can be used with greater privacy and does not enable surveillance; respect for patient agency and autonomy	General risk of coercion with penalty incentives **Incentive size:** incentives that are too small may fail to address all relevant adherence barriers; incentives that are too large may have greater risk of coercion **Incentive type:** guaranteed rewards may have greater risk of coercion than lotteries and may be more likely to “crowd-out” intrinsic patient motivation **Incentive frequency:** less frequent incentives may increase patients’ susceptibility to present-biased preferences	General risk of violating patient freedom and privacy when using individual adherence data to develop a picture of population-level adherence or to assist in contact tracing
**Direct monitoring technology (embedded sensors) (DE)**		**Most accurate:** medication ingestion is necessary for adherence detection, but patients could induce vomiting	**Most risk:** if visible, wearable hub is potentially stigmatizing; even if invisible, may enable location surveillance and identification of “problem” patients; restricted patient agency and autonomy		
**Video observation of therapy (VOT)**		**Fairly accurate:** swallowing is observed, but patients can feign ingestion or, where a human observer is required, collude to create false report	**Least risk:** can be used with greater privacy and does not enable surveillance; respect for patient agency and autonomy		
**Direct observation of therapy (DOT)**			**Most risk:** association of patient with health care worker can be stigmatizing; restricted patient agency and autonomy		
**Indirect monitoring technology (patient- and device-facilitated) (IP and ID)**		**Least accurate:** swallowing not observed; patient can place false call or remove cap without taking medication	**Least risk:** can be used with greater privacy and does not enable surveillance; respect for patient agency and autonomy		
**Recommended intervention**	Follow-up by health care workers when non-adherent SMS reminders to take medication throughout treatment Reward incentives (e.g., in-kind goods, or reductions in insurance contributions) conditional on good adherence	Maximize accuracy by minimizing opportunity for patient deception and adherence overreporting	Minimize stigmatization and intrusiveness to preserve patient agency and promote autonomy	Use reward incentives, but minimize risk of coercion by using 2-way SMS or video conferencing between patients and providers Reward value should be carefully tailored to local social and economic context (smaller value to address patient factor barriers; larger value to address non-patient factor barriers) Daily/weekly lottery	Develop population-level picture of adherence to more efficiently use resources, learn about best practices and regions where improvement is needed, and ensure timely drug restocking Strive for anonymity, thus promoting public good while minimizing restriction of individual freedom and privacy

a For additional examples, see CDC, 2012.^38^

### Accuracy of Monitoring Technologies

Health officials must have accurate knowledge of non-adherent patients in order to provide individual support and protect public health. Monitoring technologies that accurately capture adherence data in a cost-effective and minimally burdensome way are therefore of considerable relevance to public health and the health of individuals.

Each monitoring technology presents distinct technical challenges, but here we are concerned with the potential for accuracy assuming optimal technical functioning. DOT is highly accurate in monitoring adherence as it requires direct observation. However, patients could feign drug ingestion,^39^ or DOT workers may generate false reports, thus overreporting adherence, or simply not provide treatment according to DOT guidelines.^40^ VOT is subject to similar accuracy concerns,^41^ although employing facial recognition software can avoid issues associated with human observers. IP/ID faces greater accuracy challenges than DOT or VOT. IP may require placing a free call to a central server after taking medication. ID may use medication bottles that automatically transmit messages to a server following cap removal. It is relatively easy for patients to falsely report adherence by placing calls or removing pill caps without ingesting medication. In terms of accuracy, DE and DM are potentially superior to DOT, VOT, and IP/ID by requiring actual ingestion of the drugs rather than health worker or patient reports.

Direct monitoring technology through embedded sensors or metabolite testing, which require actual ingestion of drugs, are potentially more accurate than DOT and other technologies that depend on health worker or patient reports.

### Stigmatization and Intrusiveness of Monitoring Technologies

DOT is potentially stigmatizing by sometimes requiring health personnel to be present in patients’ workplaces or communities, which may distinguish patients and result in unwanted public disclosure of disease status.^42^ Because stigmatization can deter patients with TB from seeking treatment^14^ and has other serious socioeconomic implications,^42^ monitoring technologies that increase stigmatization are harmful to these individuals and counter-productive from a public health perspective. Monitoring technologies that focus unwanted attention on patients should therefore be avoided.

Monitoring technologies that focus unwanted attention on patients should be avoided.

DE that employs visibly worn wireless hubs risks stigmatizing patients. In most current forms, DE uses ingestible sensors embedded in either pills or standard pill capsules. A wearable hub that attaches to the wrist, arm, hip, or abdomen detects when capsules have been swallowed and transmits adherence data wirelessly to a central server. VOT and IP/ID are less stigmatizing by not requiring patients to publicly meet with health personnel or wear a potentially visible hub. Similarly, urinalysis-based DM would be less stigmatizing if employed in regions where people have privacy when relieving themselves.

Patients have reported that DOT feels like “doing time” and that it is “awkward” to be observed while taking treatment.^14^ DOT can be perceived as dehumanizing^43^ and may therefore contribute to self-stigma, characterized by low self-esteem and self-efficacy.^44^ Alternatively, patients have reported that VOT allowed them to retain a sense of control over their care,^28^ thus promoting autonomy, and that VOT was less intrusive than DOT given that videophone conferences last only a few minutes and can be scheduled with greater flexibility.^41^ DE that employs wearable monitoring technologies may extend perceptions of intrusive surveillance, even if worn invisibly beneath clothing.^45^ (Although there is debate in the electronic health field over the precise meanings of “intrusiveness” and “obtrusiveness,”^46^ we use the former for simplicity’s sake.) Additionally, wearable technologies could be equipped to enable location surveillance of patients by authorities in a way not possible with IP/ID, DM, and VOT, which are non-wearable, and may have detrimental effects on these grounds. Governments may use adherence data to identify problematic or expensive patients and initiate more active engagement in potentially unwelcome forms, such as compulsory detention.^47^ To varying extents, these possibilities show that tensions between particular monitoring technologies and respecting individual autonomy are possible.

VOT, IP/ID, and DM respect individual autonomy better than DOT and DE by relying on less intrusive involvement in adherence monitoring. Such technologies are potentially agency-promoting, while the others may be experienced as more controlling technologies that limit autonomy.

Video observation, indirect monitoring, and direct monitoring through metabolite testing respect patient autonomy better than DOT.

### The Use of Incentives

Incentives may be paired with mHealth monitoring technologies to enhance adherence. There is demonstrated interest in understanding whether incentives can effectively enhance TB treatment adherence within a non-mHealth context,^48^ as well as whether incentives paired specifically with mHealth monitoring can effectively enhance adherence within non-TB disease contexts.^22^ Therefore, it is possible in principle to envision the combination of incentives and mHealth monitoring to enhance TB treatment adherence, especially given that mHealth can facilitate and enable the administration of incentives in various ways.

Incentives structured as rewards generally are ethically preferable over those structured as penalties.

It might seem odd to offer follow-up, reminders, or incentives assuming that—absent external barriers, particularly structural, social, or health care service factors—patients should have a clear self-interest in taking their medication. Yet even when external barriers are not an issue, adherence rates are not ideal. For example, one study identified a 22% rate of incorrect warfarin doses to prevent blood clotting even when cost and distance were not significant adherence barriers.^22^ Behavioral economists have systematically studied the reasons for such behavior. Decision errors include powerful cognitive mechanisms such as present-biased preferences, in which short-term benefits are given disproportionate weight relative to long-term benefits.^49^ In the case of TB adherence, this phenomenon may lead patients to prioritize immediate relief from medication side effects (by not taking the medication) over the future possibility of cure, even when cure is desired. Even absent side effects, the effort required to regularly take medication can overshadow long-term gains. The challenging life circumstances often found in LMICs only exacerbate these difficulties. Incentives that offer a tangible short-term benefit can counteract these dynamics and help patients overcome such biases.^50^

Present-biased preferences may lead patients with TB to stop taking their medication because they prioritize immediate relief from medication side effects over the future possibility of cure.

For these reasons, interventions to enhance TB treatment adherence, including both DOT and mHealth approaches, may employ incentives alongside adherence monitoring, although many, including DOT, often do not do so because administering incentives can have unintended effects or be logistically challenging.^51^ Insofar as these challenges can be met, however, we discuss here the risk of coercion associated with using incentives as well as the size, type, and frequency of the incentive.

#### The Risk of Coercion

Incentives can be structured as either rewards (e.g., cash, reductions in insurance contributions, or in-kind goods) or penalties (e.g., insurance surcharges). On one account, only threats can coerce since threats indicate that an individual’s failure to behave in a desired manner will result in that individual being made worse off.^52^ Because penalties constitute threats, rewards appear ethically preferable. Still, a reward may also risk coercion insofar as the offer is one an individual could not reasonably refuse.^53^ Rewards very large in value may lead individuals to engage in behaviors that are not in their best interests. For example, patients receiving incentives for adherence may be less inclined to report side effects or other treatment-related difficulties such as an inability to work if they believe doing so may result in reduced or revoked incentives.

Policy makers should take seriously these treatment-related difficulties. Rather than remain unidirectional,^54^ mHealth interventions should connect health workers with individuals requiring extra attention by employing 2-way SMS communication or video conferencing to allow patients to provide reasons for non-adherence and still receive the incentive. Such feedback and patient assurance may enable ethical use of incentives within both DS-TB and DR-TB populations, despite the more severe side effects of DR-TB treatment. In general, allowing interaction between patient and provider may facilitate development of caring relationships that support adherence, a potential benefit of DOT that may be lost with mHealth approaches.

#### Incentive Size

Reward incentives of small value may sufficiently spur adherence in high-income countries where patient factors such as present-biased preferences are the primary obstacles to adherence (as in the case of warfarin). However, social context and structural and health care service factors may be more significant in LMICs. For example, incentives of small value may be inadequate where patients experience significant income loss during treatment or must travel long distances for drug resupply. mHealth interventions for adherence should therefore consider using incentives of large value to enhance adherence by addressing major costs of treatment, which can include direct medical and non-medical costs and lost income. It is far from straightforward to determine non-coercive incentive values, which requires considerations of effectiveness and fairness alongside country-specific circumstances. Still, there are useful benchmarks, such as average basic household expenses or daily local labor rates.^55^

Because structural and health care service factors are significant in LMICs, incentives of large value should be considered to offset major costs of TB treatment.

#### Incentive Type

Incentives can be provided with either more or less certainty, which has implications for effectively influencing motivation. Most incentives are fixed expectations whereby a reward of known value is provided whenever the qualifying activity is completed. However, incentives can be offered in less direct ways, for example, through lotteries. Here, patients who properly adhere (e.g., by ingesting all prescribed treatment doses within a specified period) receive a *chance* of winning a reward, not a guarantee. Lotteries are attractive because they can decouple behavior from the firm expectation of receiving immediate benefits for adherence, while still functioning as an effective “nudge.”^56^ Such incentives are less likely to coerce patients. Furthermore, lack of a certain reward may help avoid “crowding-out” intrinsic motivation,^56^ which is concerning for several reasons^57^ and has been observed empirically in some cases,^58^ although not universally.^59^^,^^60^

“Lotteries” in which patients have a chance of winning a reward, not a guarantee, are less likely to be coercive.

#### Incentive Frequency

Incentive frequency may impact patients’ susceptibility to present-biased preferences. For example, when considering the short-term benefit of no side effects, patients may de-prioritize the relatively long-term benefit of a small monthly or yearly reward just as they would the long-term benefit of cure. Daily incentives can counter this tendency.^50^ Lotteries can be especially useful. For example, participants in a warfarin adherence study were given a daily 1-in-5 chance of winning US$10 and a 1-in-100 chance of winning US$100, with an expected value of US$3 per day. In addition to providing feedback to patients with successful adherence, participants were also told what they would have won had they adhered, exploiting the motivating power of anticipated regret—another powerful behavioral economics principle.^22^ Providing incentives on a less frequent basis (e.g., weekly) can still have an impact, but higher frequency can increase the traction of the approach.

### Balance of Individual and Public Good

Individual adherence data can be used to better understand population-level adherence. Knowing in advance which regions have lower adherence rates can enable more efficient resource allocation such as drug restocking—insofar as there are no prohibitive supply chain or logistical obstacles—therefore reducing the risk that patients non-adhere from lack of drugs. Officials could also study these regions to develop detailed knowledge of the factors working against adherence within specific contexts. Conversely, analyzing regions with high adherence rates could reveal best practices for application elsewhere. Striving to keep individual-level adherence data anonymous, or implementing robust firewalls where this is not feasible, can help ensure that each approach serves the public good while respecting individual privacy.

Individual adherence data can be used to better understand population-level adherence.

DE that employs wearable monitoring technologies equipped with location surveillance, while potentially stigmatizing and intrusive, could alternatively assist in contact tracing and interrupting disease transmission. Policy around the use of mHealth must consider and balance the potential for promoting both individual and public good.

## THE ETHICAL BOTTOM LINE

In summary, mHealth interventions for TB treatment adherence have the potential to ethically improve on DOT in line with WHO’s End TB Strategy. In Table 2, we summarize the strengths and weaknesses of each intervention category, highlight areas of ethical concern and opportunities for ethical improvement over DOT, and provide a set of recommendations for future interventions.

Based on this analysis, we suggest urinalysis-based DM as the mHealth intervention category with the greatest potential for ethical acceptability. Assuming optimal technical functioning, DM maximizes accuracy in monitoring by most effectively restricting opportunities for deception. This model also minimizes stigmatization and intrusiveness compared with DOT and other monitoring technologies, preserving patient agency and promoting autonomy.

Of course, mHealth for TB treatment adherence will be implemented in diverse health care contexts and adherence barriers will vary widely. Urinalysis-based DM may not always be the most practical or cost-effective intervention. Where other intervention categories are more appropriate, our summary and recommendations also provide valuable ethical guidance. The ethical strengths and weaknesses of each mHealth intervention category must always be balanced with the specific barriers faced by patients, as well as the practical realities of implementing public health programs such as cost-effectiveness. Difficult choices regarding trade-offs are inevitable, but ethical acceptability should be a critical component of these debates.

## CONCLUSION

Controlling TB is urgent. While proper treatment adherence is critical to TB control, barriers to adherence are significant and diverse. mHealth constitutes an emerging field with particular promise to address such barriers, thus improving individual and population health and health systems efficiency. The ethical framework established here is intended to help with developing ethical mHealth interventions for TB adherence by flagging key ethical issues that need to be considered in planning, implementing, and evaluating programs.

## Notes

**Peer Reviewed**
